# Defending Against Randomly Located Eavesdroppers by Establishing a Protecting Region

**DOI:** 10.3390/s20020438

**Published:** 2020-01-13

**Authors:** Tao Li, Chaozheng Xue, Yongzhao Li, Octavia A. Dobre

**Affiliations:** 1 State Key Laboratory of Integrated Service Network, Xidian University, Xi’an 710071, China; taoli@xidian.edu.cn (T.L.); yzli@mail.xidian.edu.cn (Y.L.); 2 Department of Electrical and Computer Engineering, Memorial University, St. John’s, NL A1B 3X5, Canada; odobre@mun.ca

**Keywords:** physical layer security, eavesdropper, outage probability, insecure region

## Abstract

The security problem in wireless sensor networks faces severe challenges, due to the openness of the sensor network channel and the mobility and diversity of the terminals. When facing randomly located eavesdroppers, the situation is much more complex. This paper studies the security performance of a wireless sensor network where randomly located passive and active eavesdroppers are both considered. Compared to a passive eavesdropper, an active eavesdropper can perform both eavesdropping and malicious jamming simultaneously in a wireless sensor network. Based on beamforming and artificial noise (AN), we propose a practical way to defend against the eavesdropper by establishing a protecting region. An appropriate metric, the hybrid outage probability, which takes both the transmission outage probability and the secrecy outage probability into consideration, is utilized to evaluate the security performance. In addition, the concept of safe transmission range is defined to evaluate the security performance. Simulation results are provided to depict the insecure region and verify the harm of the active eavesdropper to the transmission in the wireless sensor network.

## 1. Introduction

Along with the emergence of numerous wireless devices and various wireless services, wireless security has become a critical design issue in the implementation and operation of wireless sensor networks [1,2,3,4]. Against this background, physical layer security (PLS) has been receiving great research attention [5]. Compared to traditional key-based cryptographic techniques applied to upper layers which can be deciphered, PLS which exploits the channel characters to enhance security, can safeguard wireless data transmissions without requiring secret keys and complex algorithms [6,7,8]. The main design goal of PLS is to increase the performance difference between the link of the legitimate receiver and that of the eavesdropper by using well-designed transmission schemes in the wireless sensor networks [9]. In particular, beamforming and artificial noise (AN) are exploited to improve the security performance [10,11,12,13]. Most works assume that eavesdroppers work with a passive way in the wireless sensor networks. However, there are also active eavesdroppers who can eavesdrop information in a more "smart" way. An active eavesdropper, which can perform both eavesdropping and malicious jamming simultaneously, brings an intractable challenging security problem [14,15,16,17,18,19,20,21,22,23,24].

One of the active eavesdropping methods is pilot contamination in a wireless sensor network. The eavesdropper attacked the training phase to cause pilot contamination in wireless communication to improve its eavesdropping performance [14,15,16]. Another active eavesdropping method is jamming. In the presence of an active eavesdropper, the authors in [17,18] calculated an optimal power allocation to improve the security performance of transmission. In [19,20], the properties of the game equilibrium were exploited to design a transmission strategy and a jamming strategy, where the eavesdropper took action first as the leader and the legitimate user acts as the follower in the wireless network. In [21], a three-stage Stackelberg game approach was proposed to improve the security performance under the competitions among the transmitter, relays and active eavesdropper. Finding the Stackelberg equilibrium of the scheme, and the legitimate users can achieve cooperative communication to improve the secrecy capacity and to defend against full-duplex active eavesdropping attacks. A novel transmission outage constrained scheme for both reliability and security was proposed to evaluate the secrecy performance and to gain valuable design insights in [22]. An optimal relay selection scheme was developed to improve the security performance with an active eavesdropper in cooperative wireless networks in [23,24].

These works mainly focused on adjusting the transmission strategies to obtain a better performance under the effect of self interference at the active eavesdropper and neglected the location of the eavesdropper in the wireless sensor networks. However, in practice, the location of the eavesdropper is unknown and this can change its location to cause severe interference for the transmission with small power. In this case, the above transmission strategies do not work well, which brings an intractable challenge for the transmission. Hence, the location of the active eavesdropper, as a vital parameter, has to be considered.

Under the assumption that the eavesdropper is passive, the authors in [25,26] revealed that the uncertainty on the location of the eavesdropper should be seriously taken into account for deploying a wireless sensor network system. A piecewise function was proposed to approximate the line-of-sight (LoS) probability for the air-to-ground links, which provides a better approximation than using the existing sigmoid-based fitting under randomly located unmanned aerial vehicle eavesdroppers [27]. The secrecy outage analysis of the randomly located eavesdroppers, which act independently and collude to intercept the transmitted message, was studied in [28]. The insecure region refers to a geographical area where certain security metrics such as average secrecy capacity and secrecy outage probability are not satisfied [29,30,31,32,33,34]. In [29], both the legitimate receiver and transmitter generated AN to impair the eavesdropper’s channel, and the insecure region was defined by the average secrecy capacity to characterise the security performance when the eavesdropper’s channel was unknown. A concept of outage secrecy region to evaluate the secrecy performance from a geometrical perspective was proposed in [30], where the legitimate receiver generated AN to impair the eavesdropper’s channel. However, the approximate secrecy capacity was not accurate to define the insecure region. Then, outage probability, as a more appropriate metric, was exploited to determine the insecure region [31,32,33,34]. Authors examined the impact of the unmanned aerial vehicle jamming power and its three-dimensional spatial deployment on the outage probability of the legitimate receiver and the intercept probability of the eavesdropper. The security region was defined by the intercept probability [31]. In [32], one relay node in the sensor networks can improve the security by decreasing the area in which the eavesdropper can reside and listen to the information transmitted to the destination. This region was called vulnerability region with its characterization. In [33,34], with the design of AN, high outage performance around the around the transmitter was achieved.

Inspired by the above works, we propose a practical way to defend against an active eavesdropper by establishing a protecting region to restrict the location of an active eavesdropper in a wireless sensor network. Since the eavesdropper is able to emit a jamming signal to interfere with the transmission, the traditional metrics are not appropriate in this case, and a new metric, namely hybrid outage probability, is exploited to evaluate the security performance. Specifically, we derive the expression of the hybrid outage probability which takes both the transmission outage probability and the secrecy outage probability into consideration both for active and passive eavesdropper. Based on it, the insecure region is defined to confront the eavesdropper. And the concept safe transmission range, as a valuable indicator, is proposed. In our system, the AN is generated from receiver. This method has the following advantages. (a) The CSI is not needed by Alice, so there is no feedback channel and thus the bandwidth resource is saved; (b) The AN can be generated by either multiple antennas or a single antenna, which is more practical than the existing AN methods which need multiple antennas at the transmitter; (c) It is particularly useful when the receiver has a stronger ability than the transmitter; (d) It is efficient if Eves are located around Bob [29,30,35]. Our analysis can be used in various practical sensor networks to provide valuable basis for establishing the protecting region and achieve secure transmission.

## 2. System Model

**Figure 1:**
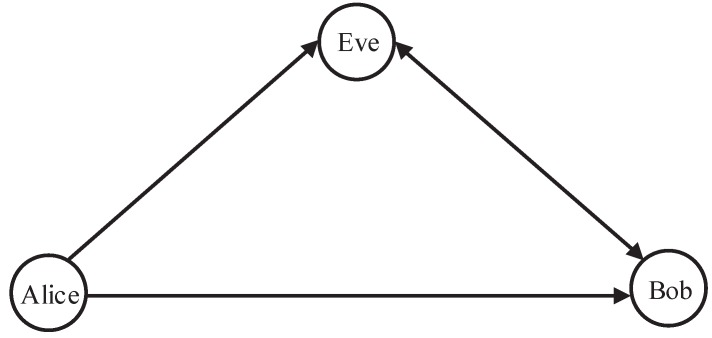
System model.

We consider a multiple-input single-output (MISO) system in the presence of a full-duplex active eavesdropper (We assume that the distribution of location of eavesdropper is a homogeneous Poisson point process, and the eavesdropper works independently. In this case, an eavesdropper with changeable location can be popularized to multiple randomly located eavesdroppers.), as shown in Figure 1. Alice with $N_{a}$ uniform linear array (ULA) antennas aims to transmit a confidential signal to Bob. In order to enhance the secrecy performance, beamforming is utilized at Alice. Bob and randomly located Eve are both equipped with one receiving antenna and one transmitting antenna [17,18]. Bob simultaneously receives the signal from Alice and emits the AN signal omnidirectionally to confuse the potential eavesdropper, while Eve simultaneously eavesdrops the signal from Alice and transmits jamming signal to interfere with the transmission. Since the full duplex capability at Bob, we assume the cancellation is not perfect. $h_{bb}$ is the residual self interference after the self-interference cancellation. It is often assumed that the self-interference can be significantly suppressed [36], so that $h_{bb}$ can be regarded as an independent Rayleigh distributed variable [37]; and $\rho \in \left[0,1\right]$ is the linear residual self-interference coefficient. As for Eve, since the legitimate users cannot obtain the information of Eve, the worst case that Eve’s self-interference can be eliminated perfectly is considered for the robust design.

The main channel and the wiretap channel can be expressed as $1\times N_{a}$ vector $h_{ab}$ and $h_{ae}$, respectively. Besides, the scalar $h_{be}$ and $h_{eb}$ represent the channels from Bob to Eve and Eve to Bob, respectively. All channels are assumed to be the flat Rayleigh fading. We assume that the Eve’s CSI and location are unknown to both Alice and Bob and the full CSI of Bob is available for Alice. The received signals at Bob and Eve are respectively expressed as

(1) $$y_{b}^{\mathrm{act}}=\sqrt{\phi P}h_{ab}w_{a}x_{a}+\sqrt{P_{e}}h_{eb}x_{e}+\sqrt{\rho \left(1-\phi \right)P}h_{bb}x_{b}+n_{b}$$

and

(2) $$y_{e}=\sqrt{\phi P}h_{ae}w_{a}x_{a}+\sqrt{\left(1-\phi \right)P}h_{be}x_{b}+n_{e},$$

where $w_{a}$ represents the $N_{a}\times 1$ beamforming vector at Alice, and the superscript ${\left[\cdot \right]}^{H}$ represents Hermitian conjugate. Under the assumption that perfect CSI of Bob is assumed to be known for Alice, the optimal beamforming is designed as wa=habHhabHhabhab to enhance the receiving performance of Bob [38]. The confidential signal from Alice, the AN from Bob, and the jamming signal from Eve are respectively denoted by scalar $x_{a}$, $x_{b}$, and $x_{e}$ with unit power, i.e., $E\left[{\left|x_{a}\right|}^{2}\right]=E\left[{\left|x_{b}\right|}^{2}\right]=E\left[{\left|x_{e}\right|}^{2}\right]=1$. The total transmission power is denoted by *P*, which includes the confidential signal power from Alice and the AN power from Bob; and ϕ is the power allocation factor between the confidential signal from Alice and the AN signal from Bob. The jamming signal power from Eve is denoted by $P_{e}$. $n_{b}$ and $n_{e}$ are additive white Gaussian noises with powers $\sigma_{b}^{2}$ and $\sigma_{e}^{2}$, respectively. As our system model is also compatible with the passive Eve, i.e., when $P_{e}=0$, Eve becomes passive. Then, the received signal at Bob is

(3) $$y_{b}^{\mathrm{pas}}=\sqrt{\phi P}h_{ab}w_{a}x_{a}+\sqrt{\rho \left(1-\phi \right)P}h_{bb}x_{b}+n_{b}.$$

## 3. Insecure Region Analysis

In this section, the correctness of the hybrid outage probability for active Eve is verified firstly. Subsequently, the expression of the hybrid outage probability is derived. Based on it, the insecure region and safe transmission range are defined to evaluate the security performance.

### 3.1. Hybrid Outage Probability

From (1) and (2), the signal-to-interference-noise ratio (SINR) at Bob and active Eve can be respectively calculated as (This paper aims to establish the insecure region to defend against the active eavesdropper and achieve a higher security performance. The self-interference is beyond our main focus. This is modeled as a variable depending on the ability of Bob, according to [18,22]. The value of self-interference is changed with ρ.)

(4) $$\gamma_{b}^{\mathrm{act}}=\frac{\phi P_{a}\lambda d_{ab}^{-\beta }{∥h_{ab}w_{a}∥}^{2}}{P_{e}\lambda d_{be}^{-\beta }{\left|h_{eb}\right|}^{2}+\rho \left(1-\phi \right)P+\sigma_{b}^{2}}$$

and

(5) $$\gamma_{e}=\frac{\phi P\lambda d_{ae}^{-\beta }{∥h_{ae}w_{a}∥}^{2}}{\left(1-\phi \right)P\lambda d_{be}^{-\beta }{\left|h_{be}\right|}^{2}+\sigma_{e}^{2}},$$

where $∥\cdot ∥$ denotes the Euclidean norm, $d_{ab}$, $d_{ae}$, and $d_{be}$ represent the distances between Alice and Bob, Alice and Eve, Bob and Eve, respectively, λ is a constant which depends on the propagation model and carrier frequency, β≥2 is the path-loss exponent. When Eve is passive, from (3), the SINR at Bob is

(6) $$\gamma_{b}^{\mathrm{pas}}=\frac{\phi P_{a}\lambda d_{ab}^{-\beta }{∥h_{ab}w_{a}∥}^{2}}{\rho \left(1-\phi \right)P+\sigma_{b}^{2}}.$$

The secrecy capacity is expressed as [29,30]

(7) $$C_{s}=\left\{\begin{array}{cc} C_{b}-C_{e}, & under\;C_{b}>C_{e};\\ 0, & under\;C_{b}\leq C_{e}, \end{array}\right.$$

where $C_{b}=\log_{2}\left(1+\gamma_{b}\right)$ is the main channel capacity between Alice and Bob. $C_{e}=\log_{2}\left(1+\gamma_{e}\right)$ is the wiretap channel capacity between Alice and Eve. Since the CSI of the wiretap channel is unavailable, the instantaneous secrecy capacity is unobtainable. Thus, the outage probability is a more suitable metric for our system.

If the secrecy transmission rate is assumed to be $R_{s}$, the entire event space of communication can be divided into three mutually exclusive events [39]:

- Transmission outage event occurs when $C_{b}\leq R_{s}$. In this case, we find $C_{e}=C_{b}-R_{s}<0$, which conflicts with the fact that $C_{e}>0$. As such, $R_{s}$ is not supported by the main channel and Alice can not transmit a signal.
- Secrecy outage event occurs when $C_{s}<R_{s}$ and $C_{b}>R_{s}$. In this case, as some information on the confidential signal can be known by Eve, perfect secrecy cannot be achieved.
- Secure transmission event occurs when $C_{s}\geq R_{s}$. In this case, perfect secrecy can be guaranteed.

In the conventional scenario where Eve is passive, the main performance metric is secrecy outage probability. However, in our system, Eve can emit malicious interference to destroy the transmission, which causes the transmission outage event. In this case, the secrecy outage probability cannot evaluate the performance comprehensively. Hence, we adopt the hybrid outage probability as performance metric, as follows

(8) $$P_{\mathrm{ho}}\left(\theta ,d_{ae},d_{be}\right)=P_{\mathrm{to}}\left(\theta ,d_{ae},d_{be}\right)+P_{\mathrm{so}}\left(\theta ,d_{ae},d_{be}\right),$$

where $P_{\mathrm{to}}\left(\theta ,d_{ae},d_{be}\right)$ represents the transmission outage probability and $P_{\mathrm{so}}\left(\theta ,d_{ae},d_{be}\right)$ represents the secrecy outage probability. Meanwhile, the hybrid outage probability is also applicable to passive Eve.

In order to obtain the expressions of the outage probabilities, we present the statistics of $\gamma_{b}^{\mathrm{act}}$ and $\gamma_{e}$. From the right hand side of (4), we find the numerator follows a chisquared distribution since ${∥h_{ab}∥}^{2}$ is a sum of the squares of $N_{a}$ independent Gaussian random variables; and the denominator follows an exponential distribution. Meanwhile, as the numerator and denominator are independent, we apply

(9) $$F{}_{\frac{X}{Y+1}}\left(\gamma \right)=\mathrm{Pr}\left(\frac{x}{y+1}<\gamma \right)=\int_{0}^{\infty }\left(y+1\right)f_{X}\left(\gamma \left(y+1\right)\right)f_{Y}\left(y\right)dy$$

to obtain the cumulative distribution functions (CDF) of $\gamma_{b}^{\mathrm{act}}$ as

(10) $$F_{\gamma_{b}}^{\mathrm{act}}\left(\gamma \right)=1-\frac{1}{k_{2}}\exp\left(-\frac{\gamma }{k_{1}}\right)\sum_{n=1}^{N_{a}}\frac{1}{\left(n-1\right)!}{\left(\frac{\gamma }{k_{1}}\right)}^{n-1}\sum_{m=0}^{n-1}\frac{\left(n-1\right)!}{\left(n-m-1\right)!}{\left(\frac{1}{k_{2}}+\frac{\gamma }{k_{1}}\right)}^{-\left(m+1\right)},$$

where

$$k_{1}=\frac{\lambda d_{ab}^{-\beta }\phi P}{\rho \left(1-\phi \right)P+\sigma_{b}^{2}}$$

and

$$k_{2}=\frac{\lambda d_{be}^{-\beta }P_{e}}{\rho \left(1-\phi \right)P+\sigma_{b}^{2}}.$$

We now derive the CDF of $\gamma_{e}$. Due to the fact that the beamforming vector $w_{a}$ at Alice is independent from eavesdropper’s channel $h_{ae}$, the denominator follows exponentially distributed; and the numerator is also exponentially distributed. Similarly, the numerator and denominator are independent. With the help of (9), the CDF of $\gamma_{e}$ is

(11) $$F_{\gamma_{e}}\left(\gamma \right)=1-\frac{k_{3}}{k_{4}\gamma +k_{3}}\exp\left(-\frac{\gamma }{k_{3}}\right),$$

where

$$k_{3}=\frac{\lambda d_{ae}^{-\beta }\phi P}{\sigma_{e}^{2}}$$

and

$$k_{4}=\frac{\lambda d_{be}^{-\beta }\left(1-\phi \right)P}{\sigma_{e}^{2}}.$$

According to the definition of the transmission outage event with active Eve, we have

(12) $$\begin{array}{c} P_{\mathrm{to}}^{\mathrm{act}}\left(\theta ,d_{ae},d_{be}\right)=\mathrm{Pr}\left(0<C_{b}\leq R_{s}\right)=\mathrm{Pr}\left(0<\gamma \leq 2^{R_{s}}-1\right)=F_{\gamma_{b}}^{\mathrm{act}}\left(2^{R_{s}}-1\right)\\ =1-\frac{1}{k_{2}}\exp\left(-\frac{2^{R_{s}}-1}{k_{1}}\right)\sum_{n=1}^{N_{a}}\frac{1}{\left(n-1\right)!}{\left(\frac{2^{R_{s}}-1}{k_{1}}\right)}^{n-1}\sum_{m=0}^{n-1}\frac{\left(n-1\right)!}{\left(n-m-1\right)!}{\left(\frac{1}{k_{2}}+\frac{2^{R_{s}}-1}{k_{1}}\right)}^{-\left(m+1\right)}. \end{array}$$

The hybrid outage probability in (8) can be re-expressed as

(13) $$\begin{array}{cc} P_{\mathrm{ho}}\left(\theta ,d_{ae},d_{be}\right) & =P_{\mathrm{to}}\left(\theta ,d_{ae},d_{be}\right)+P_{\mathrm{so}}\left(\theta ,d_{ae},d_{be}\right)\\ \; & =\mathrm{Pr}\left(C_{b}\leq R_{s}\right)+\mathrm{Pr}\left(C_{b}<R_{s}+C_{e},C_{b}>R_{s}\right)\\ \; & =\mathrm{Pr}\left(0<C_{b}<R_{s}+C_{e}\right)\\ \; & =\mathrm{Pr}\left(0<\gamma_{b}<2^{R_{s}}\left(1+\gamma_{e}\right)-1\right)\\ \; & =\int_{0}^{\infty }\int_{0}^{2^{R_{s}}\left(1+\gamma_{e}\right)-1}f_{\gamma_{b}}\left(\gamma_{b}\right)f_{\gamma_{e}}\left(\gamma_{e}\right)d\gamma_{b}d\gamma_{e}\\ \; & =\int_{0}^{\infty }f_{\gamma_{e}}\left(\gamma_{e}\right)F_{\gamma_{b}}\left(2^{R_{s}}\left(1+\gamma_{e}\right)-1\right)d\gamma_{e}. \end{array}$$

Then, from (11), the probability density function (PDF) of $\gamma_{e}$ can be derived as

(14) $$f_{\gamma_{e}}\left(\gamma \right)=\left(\frac{k_{3}k_{4}}{{\left(k_{3}+k_{4}\gamma \right)}^{2}}+\frac{1}{k_{3}+k_{4}\gamma }\right)\exp\left(-\frac{\gamma }{k_{3}}\right).$$

By substituting (9) and (13) into (12), the hybrid outage probability in the presence of an active Eve is derived as

(15) $$\begin{array}{cc} P_{\mathrm{ho}}^{\mathrm{act}}\left(\theta ,d_{ae},d_{be}\right)= & 1-\frac{1}{k_{2}}\int_{0}^{\infty }\exp\left(-\left(\frac{\gamma_{e}}{k_{3}}+\frac{2^{R_{s}}\left(1+\gamma_{e}\right)-1}{k_{1}}\right)\right)\\ \; & \sum_{n=1}^{N_{a}}{\left(\frac{2^{R_{s}}\left(1+\gamma_{e}\right)-1}{k_{1}}\right)}^{n-1}\sum_{m=0}^{n-1}\frac{1}{\left(n-m-1\right)!}{\left(\frac{1}{k_{2}}+\frac{2^{R_{s}}\left(1+\gamma_{e}\right)-1}{k_{1}}\right)}^{-\left(m+1\right)}\\ \; & \left(\frac{k_{3}k_{4}}{{\left(k_{4}\gamma_{e}+k_{3}\right)}^{2}}+\frac{1}{k_{4}\gamma_{e}+k_{3}}\right)d\gamma_{e}. \end{array}$$

It is clear that the secrecy outage probability can be calculated through (15) and (12) with the help of (8).

From (6), ${∥h_{ab}∥}^{2}$ is a sum of the squares of $N_{a}$ independent Gaussian random variables, the CDF of $\gamma_{b}^{\mathrm{pas}}$ is

(16) $$F_{\gamma_{b}}^{\mathrm{pas}}\left(\gamma \right)=1-\exp\left(-\frac{\gamma }{k_{1}}\right)\sum_{n=0}^{N_{a}-1}\frac{1}{n!}{\left(\frac{\gamma }{k_{1}}\right)}^{n}$$

The analysis of the outage probability expressions with passive Eve are similar. The transmission outage probability and the hybrid outage probability are derived, as follows

(17) $$P_{\mathrm{to}}^{\mathrm{pas}}\left(\theta ,d_{ae},d_{be}\right)=1-\exp\left(-\frac{2^{R_{s}}-1}{k_{1}}\right)\sum_{n=0}^{N_{a}-1}\frac{1}{n!}{\left(\frac{2^{R_{s}}-1}{k_{1}}\right)}^{n}$$

and

(18) $$\begin{array}{cc} P_{\mathrm{ho}}^{\mathrm{pas}}\left(\theta ,d_{ae},d_{be}\right)= & 1-\int_{0}^{\infty }\exp\left(-\left(\frac{\gamma_{e}}{k_{3}}+\frac{2^{R_{s}}\left(1+\gamma_{e}\right)-1}{k_{1}}\right)\right)\\ \; & \left(\frac{k_{3}k_{4}}{{\left(k_{4}\gamma_{e}+k_{3}\right)}^{2}}+\frac{1}{k_{4}\gamma_{e}+k_{3}}\right)\sum_{n=0}^{N_{a}-1}\frac{1}{n!}{\left(\frac{2^{R_{s}}\left(1+\gamma_{e}\right)-1}{k_{1}}\right)}^{n}d\gamma_{e} \end{array}$$

where $\mathrm{Ei}\left(\cdot \right)$ is the exponential integral function [40].

### 3.2. Insecure Region and Safe Transmission Range

As mentioned above, the insecure region Ω is the set of the eavesdropper’s locations where the hybrid outage probability is larger than a given threshold denoted by 0<ε<1; this is expressed as

(19) $$\Omega =\left\{\left(\theta ,d_{ae},d_{be}\right)\left|P_{\mathrm{ho}}\left(\theta ,d_{ae},d_{be}\right)>\varepsilon \right.\right\}.$$

According to the definition of insecure region, we can establish the protecting region, where Eve is not allowed to enter to achieve secure transmission in a real communication scenario.

Eve emitting a jamming signal also brings the risk of being detected. We assume that if the jamming power from Eve increases to a certain threshold $P_{e}^{\mathrm{th}}$, it will be exposed. When Eve is located near Alice, it can intercept the confidential signal easily; when Eve is located near Bob, it can damage the legitimate transmission with small power. Hence, not only the region around Alice but also around Bob is insecure. When the jamming signal power from Eve $P_{e}$ equals $P_{e}^{\mathrm{th}}$, the boundary of the insecure region can be obtained.

**Figure 2:**
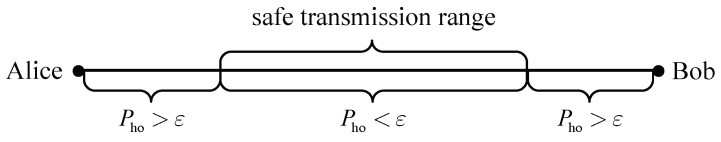
Safe transmission range.

In addition, for the worst case that Eve appears on the line between Alice and Bob, it can also obtain the power gain from Alice’s beamforming. The safe transmission range is defined by $P_{\mathrm{ho}}<\varepsilon$, as shown in Figure 2. This range is denoted by $d_{s}$, and helps delineating the circular protecting region around Alice and Bob.

## 4. Numerical Results

Simulation results are conducted to show the insecure region defined by the hybrid outage probability. Unless otherwise mentioned, the default simulation parameters are as listed in Table 1. All channels experience Rayleigh fading, i.e, λ=1. The boundary of the insecure region is obtained when the jamming signal power $P_{e}=P_{e}^{\mathrm{th}}$. The outage probabilities are calculated over 1000 trials of Monte Carlo simulations.

**Figure 3:**
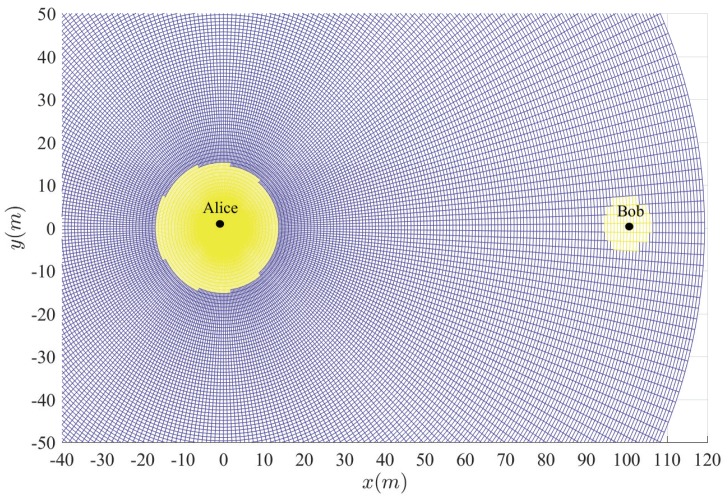
Insecure region with $P_{e}^{\mathrm{th}}=15$ dBm, $N_{a}=4$ and $\rho =10^{-8}$.

**Figure 4:**
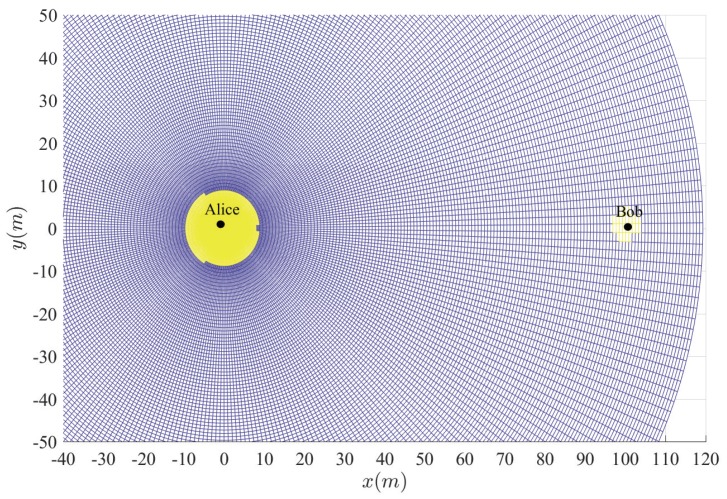
Insecure region with $P_{e}^{\mathrm{th}}=15$ dBm, $N_{a}=10$ and $\rho =10^{-8}$.

**Figure 5:**
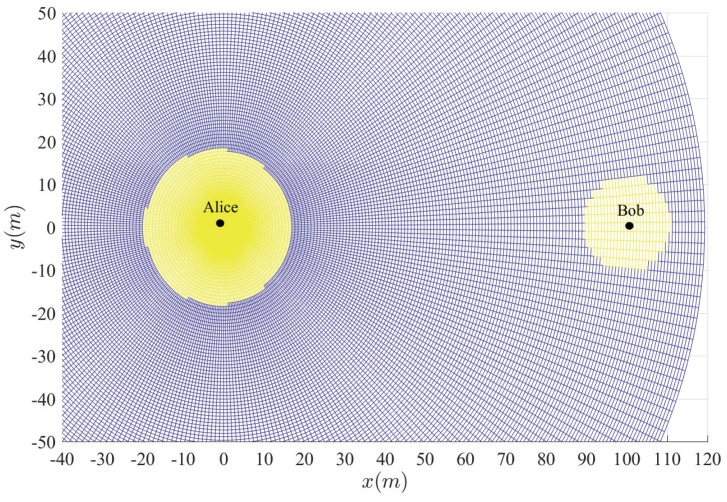
Insecure region with $P_{e}^{\mathrm{th}}=20$ dBm, $N_{a}=4$ and $\rho =10^{-8}$.

**Figure 6:**
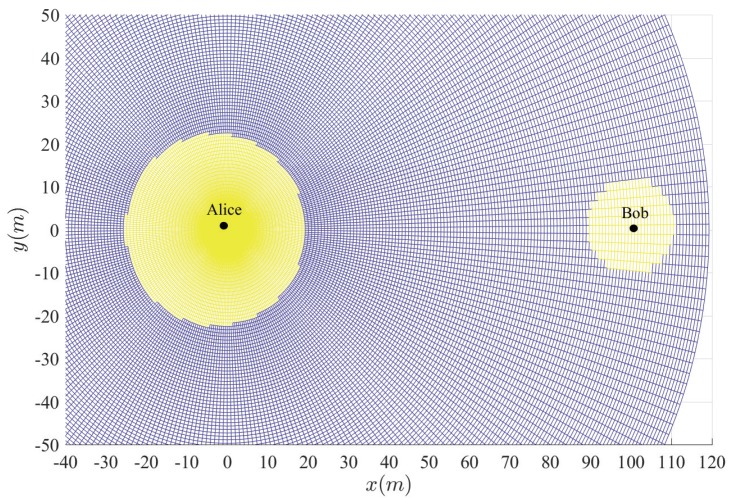
Insecure region with $P_{e}^{\mathrm{th}}=20$ dBm, $N_{a}=4$ and $\rho =10^{-6}$.

**Figure 7:**
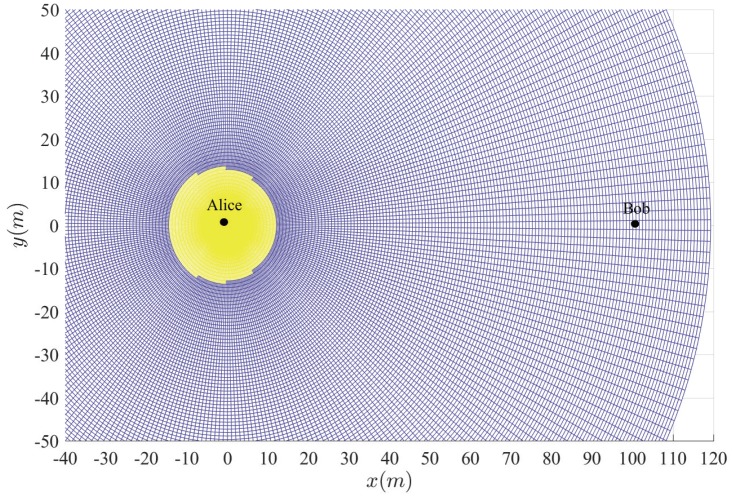
Insecure region with passive Eve and $N_{a}=4$ and $\rho =10^{-8}$.

Figure 3, Figure 4, Figure 5, Figure 6 and Figure 7 show the insecure regions with active Eve and passive Eve, respectively. The region represents the secure region where $P_{\mathrm{ho}}<\varepsilon$, while the yellow region represents the insecure region where $P_{\mathrm{ho}}>\varepsilon$. Compared with the passive Eve in Figure 7, one can see that the active Eve increases the insecure region, and the region around Bob is also insecure. Since the jamming signal from Eve can interfere with Bob, which makes the insecure region also look approximately like a disc around Bob. On the other hand, the confidential signal is transmitted from Alice, Eve appears around Alice can eavesdrop the confidential signal, which makes the insecure region look approximately like a disc around Alice. As for the active Eve in Figure 3 and Figure 4, when a large number of antennas are applied in Alice, the size of insecure region will diminish. Because the multi-antenna gain makes Bob receive the confidential signal more easily. Although it also benefits to the eavesdropper to eavesdrop the confidential signal, the gain from beamforming increases the performance difference between the link of the legitimate receiver and that of the eavesdropper, and from Figure 3 and Figure 5, when $P_{e}^{\mathrm{th}}$ increases, the insecure region enlarges, since Eve can interfere with the transmission at a further location. It is noted that the insecure region enlarges when ρ increases from Figure 3 and Figure 6 since that the AN from Bob effects itself much more. When the insecure region is determined, the secure transmission can be achieved by protecting the insecure region, which is easy to conduct due to its regular circular shape.

In the following, we consider that Eve is located on the line between Alice and Bob, and the change trend between Alice and Bob is analyzed.

**Figure 8:**
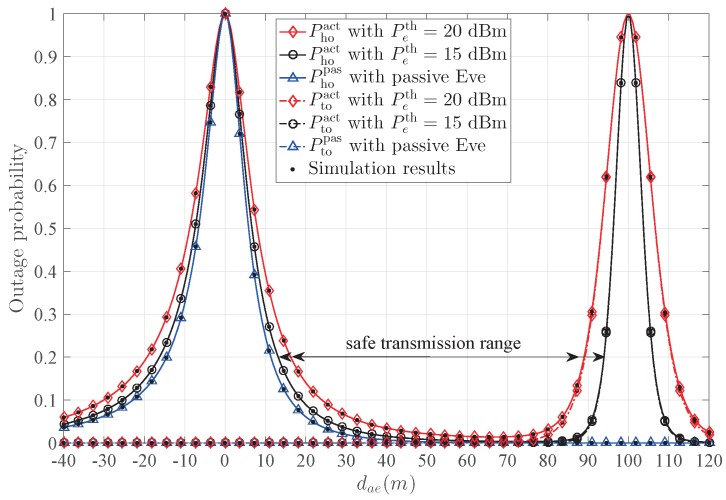
The influence of $P_{e}^{\mathrm{th}}$ on the outage probability.

Figure 8 presents the hybrid outage probability $P_{\mathrm{ho}}$ and the transmission outage probability $P_{\mathrm{to}}$ versus $d_{ae}$ with different $P_{e}^{\mathrm{th}}$. For passive Eve, $P_{\mathrm{ho}}^{\mathrm{pas}}$ decreases with the increase of $d_{ae}$, due to the fact that the power of the received confidential signal decreases and the power of the received AN from Bob increases. On the other hand, $P_{\mathrm{to}}^{\mathrm{pas}}$ is a constant, which is easily verified from (17). For active Eve, when increasing $d_{ae}$, the significant difference of $P_{\mathrm{ho}}^{\mathrm{act}}$ from passive Eve $P_{\mathrm{ho}}^{\mathrm{pas}}$ is the change around Bob. The main reason is that $P_{\mathrm{to}}^{\mathrm{act}}$ dramatically increases around Bob, since the jamming signal from Eve causes significant damage to Bob. When $P_{e}^{\mathrm{th}}$ decreases, one notices that the width of the peak around Bob decreases, which means that the insecure region around Bob diminishes, and the safe transmission range $d_{s}$ increases. According to the safe transmission range, the circular protecting region around Alice and Bob can be conducted.

**Figure 9:**
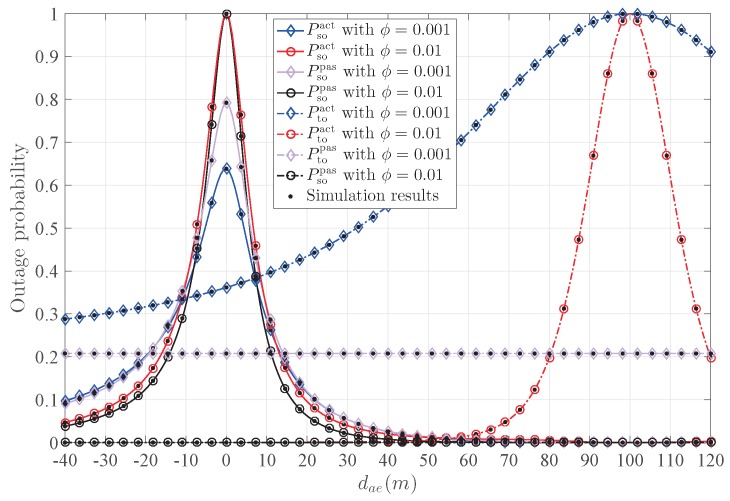
The influence of ϕ on the outage probability.

Figure 9 depicts the transmission outage probability $P_{\mathrm{to}}$ and the secrecy outage probability $P_{\mathrm{so}}$ versus $d_{ae}$ with different ϕ. As ϕ increases, $P_{\mathrm{so}}$ increases while $P_{\mathrm{to}}$ decreases; this is because increasing the power of the confidential signal is beneficial to the establishment of transmission between Alice and Bob, but also increases the risk of eavesdropping. Note that $P_{\mathrm{to}}$ represents the reliability of the transmission. Thus, a reasonable trade-off between transmission reliability and security should be considered. Meanwhile, the variation trend of $P_{\mathrm{so}}$ is the same for both active and passive Eve, which means that the active Eve mainly interferes with the establishment of the legitimate transmission link between Alice and Bob, and it can dramatically increase $P_{\mathrm{to}}^{\mathrm{act}}$ by moving around Bob.

**Figure 10:**
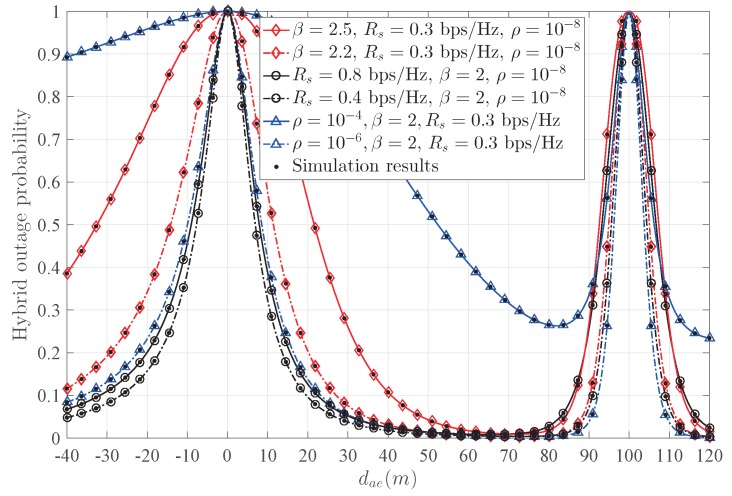
The influence of $R_{s}$, β, and ρ on the hybrid outage probability $P_{\mathrm{ho}}^{\mathrm{act}}$.

Figure 10 shows the influence of β, $R_{s}$, and ρ on the hybrid outage probability, respectively. It is clear that when β increases, the security performance decreases, since the communication condition is worse. When $R_{s}$ decreases, the security performance increases, as more power can be used to transmit the AN signal. As ρ increases, the security performance decreases, since the self-interference at Bob causes more damage to the transmission. It is worth noting that numerical results are consistent with the simulation results.

**Figure 11:**
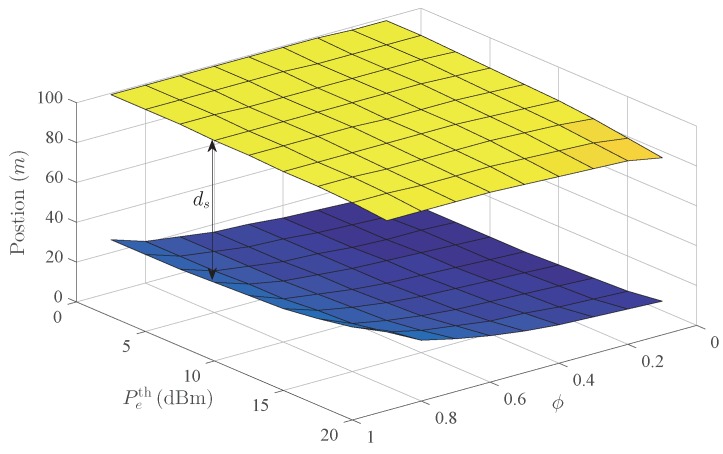
The influence of $P_{e}^{\mathrm{th}}$ and ϕ on $d_{s}$.

Figure 11 shows the influence of $P_{e}^{\mathrm{th}}$ and ϕ on $d_{s}$. The top and bottom surfaces represent the positions of the right and left endpoints of the safe transmission range, respectively, with distance $d_{s}$ between them. The insecure region around Bob corresponding to the top surface mainly depends on $P_{e}^{\mathrm{th}}$, while the insecure region around Alice corresponding to the bottom surface mainly depends on ϕ. Furthermore, when $P_{e}^{\mathrm{th}}$ increases, Eve can interfere with Bob from a further location, which causes the decrease of $d_{s}$. The increase of ϕ makes it easier to intercept, which causes the decrease of $d_{s}$ as well. Obviously, all simulation results show that the active Eve can cause a higher outage probability, and is more harmful to the secure transmission.

## 5. Conclusions

This paper has proposed a valuable way to defend against an eavesdropper by establishing the protecting region to prevent the eavesdropper from entering in the wireless sensor networks. We have analyzed the insecure region based on the metric of the hybrid outage probability which takes both the transmission outage probability and the secrecy outage probability into consideration, under the assumption that the eavesdropper is passive and active. Subsequently, the hybrid outage probability expressions have been derived to define the insecure region. The safe transmission range, as an effective indicator, has been defined to conduct the circular protecting region around transceiver. The analysis of the insecure region can also be integrated with existing transmission strategies to achieve a higher security performance in wireless sensor networks.

## Figures and Tables

**Table 1:** Simulation Parameters.

Parameters	Values
Number of antennas in Alice $N_{a}$	4
Distance between Alice and Bob $d_{ab}$	100 m
Total transmission power *P*	10 W
Power allocation factor ϕ	0.1
Jamming signal power $P_{e}^{\mathrm{th}}$	15 dBm
Secrecy transmission rate $R_{s}$	0.3 bps/Hz
Threshold ε	0.2
Path loss exponent β	2
Linear residual coefficient ρ	$10^{-8}$
